# The value of an MRI‐based radiomics model in predicting the survival and prognosis of patients with extrahepatic cholangiocarcinoma

**DOI:** 10.1002/cam4.6832

**Published:** 2024-01-08

**Authors:** Limin Wang, Jiong Liu, Yanyan Zeng, Jian Shu

**Affiliations:** ^1^ Department of Radiology The Affiliated Hospital of Southwest Medical University Luzhou Sichuan China; ^2^ Nuclear Medicine and Molecular Imaging Key Laboratory of Sichuan Province Luzhou Sichuan China

**Keywords:** extrahepatic cholangiocarcinoma, MRI, radiomics, survival analysis

## Abstract

**Objectives:**

The study aimed to establish radiomics models based on magnetic resonance imaging (MRI) multiparameter images to predict the survival and prognosis of patients with extrahepatic cholangiocarcinoma (ECC).

**Methods:**

Seventy‐eight patients with ECC confirmed by pathology were collected retrospectively. The radiomics model_a/b/c were constructed based on the 1/2/3‐year survival of patients with ECC. The best texture features were selected according to postoperative survival time and ECC patient status to calculate the radiomics score (Rad‐score). A cutoff value was selected, and patients were divided into high‐risk and low‐risk groups.

**Results:**

Model_a, model_b, and model_c were used to predict 1‐, 2‐, and 3‐year postoperative survival rates, respectively. The area under the curve values in the training and test groups were 1.000 and 0.933 for model_a, 0.909 and 0.907 for model_b, 1.000 and 0.975 for model_c, respectively. The survival prediction model based on the Rad‐score showed that the postoperative mortality risk differed significantly between risk groups (*p* < 0.0001).

**Conclusions:**

The MRI radiomics model could be used to predict the survival and prognosis of patients with ECC.

## 1 INTRODUCTION

Cholangiocarcinoma (CCA) is the second most common primary hepatobiliary malignant tumor after hepatocellular carcinoma, accounting for 15%–20% of primary hepatobiliary malignant tumors and 3% of all gastrointestinal malignant tumors.^1^ According to the seventh and eighth edition of TNM staging system of the American Cancer Society, CCA is divided into intrahepatic cholangiocarcinoma (ICC) and extrahepatic cholangiocarcinoma (ECC) according to its location of onset. Extrahepatic cholangiocarcinoma is further divided into hilar cholangiocarcinoma (PCC) and distal cholangiocarcinoma (DCC).^2^ ECC accounts for approximately 80% of all CCAs.^3^ ECC is a relatively rare malignant tumor with an extremely poor prognosis. During the development of ECC, patients may show painless jaundice or abdominal pain, emaciation, and other symptoms. At the time of symptom development, ECC has invaded adjacent organs or shows distant metastasis.^4^ The average overall survival (OS) time of untreated CCA patients after the onset of clinical symptoms is <6 months.^5^

MRI is currently the most accurate and noninvasive imaging method for the diagnosis of CCA.^6^ Imaging sequences with specific parameters show high sensitivity and specificity for the detection of lymph node metastasis, and they are essential for the selection of clinical treatment.^7^ However, in many ECC patients who are considered resectable on imaging, the tumor tissue cannot be completely removed during surgery or the postoperative pathological examination indicates that the tumor is highly malignant. The survival prognosis of these patients who received ineffective resection is often poor.^8^ An accurate and quantitative method to evaluate the prognosis of patients and predict the postoperative survival before surgery is currently lacking.

The rise of artificial intelligence provides this possibility. Radiomics allows the acquisition of high‐throughput texture features from images that cannot be recognized by human eyes.^9^ Statistical analysis and further study of these texture features can provide heterogeneous information of certain types of tumors, which may facilitate predicting the prognosis of these patients.^10^ Studies have addressed the application of imaging to predict the pathological stage^11^ and lymph node metastasis^12^ of patients with ECC before surgery, which may predict the prognosis of ECC to a certain extent. However, methods to directly predict the survival prognosis of patients with ECC have not been reported to date. The purpose of this study was to establish an imaging model based on MRI to predict the postoperative survival time and survival risk of patients with ECC before surgery.

## 2 MATERIALS AND METHODS

### 2.1 Patients

Between February 2012 and July 2020, patients diagnosed with ECC (including pCCA and dCCA) by histopathology in the Affiliated Hospital of Southwest Medical University were retrospectively analyzed. All patients underwent radical resection of ECC or pancreaticoduodenectomy, with the specific surgical procedure depending on the location of the tumor.

### 2.2 Inclusion and exclusion criteria

Inclusion criteria were as follows: ① Patients with ECC confirmed by histopathology after operation, and ② patients who underwent MRI examination within 4 weeks before surgery. Exclusion criteria were as follows: ① Lack of qualified MRI images before surgery (incomplete sequence or poor image quality), ② preoperative arterial embolization or other palliative treatment for ECC, ③ small tumor volume (short diameter <0.5 cm) or the tumor foci cannot be identified on MRI images, and ④ lost to follow‐up after surgery (lost to follow‐up immediately after surgery or <1 year until the last follow‐up).

### 2.3 Follow‐up and survival time

The follow‐up was mainly carried out via telephone calls and the last outpatient registration follow‐up, and the follow‐up deadline was July 01, 2021. The telephone follow‐up questions included whether the patient survived after surgery and the specific time of death for deceased patients. For deceased patients, OS was defined as the time period from the day of surgical treatment to the day of confirmed death, marked as “dead“. For surviving patients, OS was defined as the time period from the date of surgical treatment to the date of the last follow‐up, marked as “surviving”. For predicting the 1‐, 2‐, and 3‐year survival rates of ECC patients, the shortest follow‐up time of all “surviving” patients was ≥1 year, ≥2 years, and ≥3 years, respectively. Patients who were unable to contact the hospital or were not followed‐up in the outpatient clinic for various reasons were marked as lost to follow‐up.

### 2.4 MRI scan parameters

A Philips 3.0 T superconducting whole body magnetic resonance scanner (Philips Achieva, Holland) and 16‐channel body Torso coil were used in all patients. The patients fasted for 4–8 h before examination and were trained to hold their breath before scanning. Those who could not hold their breath were scanned by breath trigger scan. The imaging protocol mainly used the following: an axial T1‐weighted high‐resolution isotropic volume excitation (THRIVE), an axial fat‐suppression turbo spin echo T2‐weighted imaging (T2WI), and an axial diffusion‐weighted imaging (DWI). The parameters of THRIVE, T2WI, and DWI sequences are shown in Table 1. The scanning range was from the top of the diaphragm to the lower edge of the horizontal part of the duodenum.

**TABLE 1:** Parameters of abdominal MRI sequences of THRIVE, T2WI, and DWI images.

Acquisition parameters	THRIVE	T2WI	DWI
Repetition time (ms)	3.1	1610	Shortest
Echo time (ms)	1.44	70	934
Flip angle(°)	10	90	90
Field of view (mm × mm)	280 × 305	280 × 305	280 × 305
Matrix size	244 × 186	176 × 201	100 × 124
Slice thickness (mm)	3	7	7
Slices	120	24	48
Number of signal averaged	1	2	4
b values (s/mm^2^)	‐	‐	0 and 800

Abbreviations: DWI, diffusion‐weighted imaging; MRI, magnetic resonance imaging; T2WI, fat‐suppression turbo spin echo T2‐weighted imaging sequence; THRIVE, T1‐weighted high‐resolution isotropic volume excitation sequence.

### 2.5 Image processing

**FIGURE 1:**
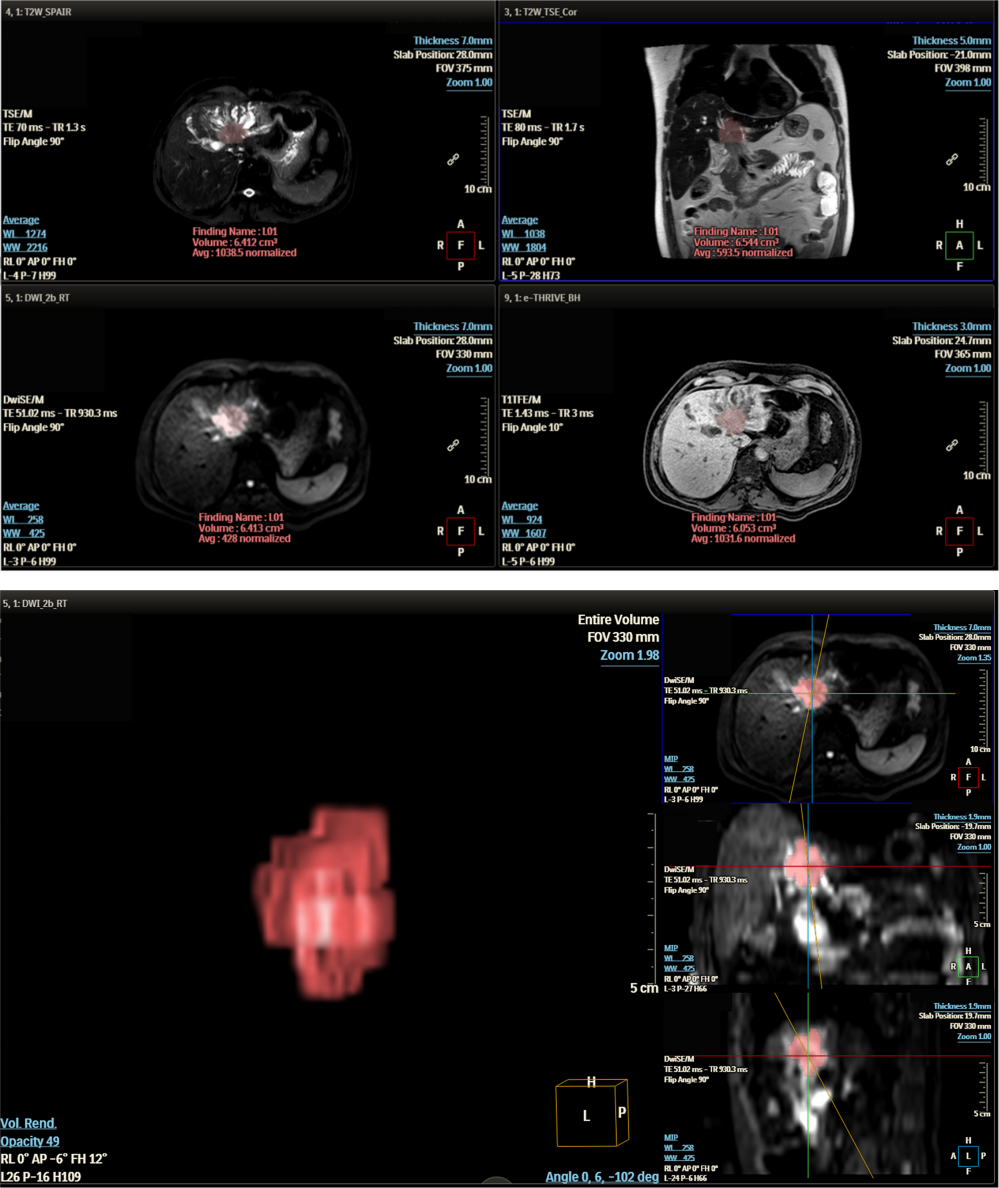
ROI sketch and VOI reconstruction map of ECC lesions.

The original MRI images of all ECC patients obtained before surgery were exported and saved in DICOM format. The images were imported into Philips IntelliSpace Discovery (ISD) 3.0.4 workstation for post‐processing. On the ISD workstation, a radiologist (with more than 3 years of experience in diagnosing abdominal MRI) sketched the region of interest (ROI) on each level of the tumor on the DWI sequence, avoiding large blood vessels and necrotic tissue as far as possible. After integration, the volume of interest (VOI) was obtained, and the VOI was replicated synchronously in T2WI and THRIVE sequences. A total of 30 patients with ECC were randomly selected and the VOIs were drawn by three radiologists (all with more than 3 years of experience in abdominal radiology diagnosis) for intraclass correlation efficient (ICC) of extracted features. The lesions with obvious disagreement regarding the delineated area were reviewed by another doctors (with more than 20 years of experience in abdominal imaging diagnosis). The image sketch and VOI reconstruction are shown in Figure 1.

### 2.6 Feature extraction

The PyRadiomic software package of the ISD workstation was used to extract the features of VOI sketched by three MRI sequences. Each VOI could extract 1199 texture features, including shape‐based feature, first‐order feature, gray‐level co‐occurrence matrix, gray‐level run length matrix, and gray‐level size zone matrix. A total of 3597 features can be extracted from each patient.

### 2.7 Prediction model establishment

#### 2.7.1 Establishment of survival prediction models

FeAtureExplorer (FAE, https://github.com/salan668/FAE) was used for the model. According to the postoperative survival time of 1, 2, and 3 years as the grouping criteria, three predictive models were established. The samples were divided randomly, and a training set and test set were established at a ratio of 8:2. The test set was not used for the training and construction of the model, and was only involved in the evaluation of the model effect. The Synthetic Minority Oversampling Technique (SMOTE) was used to balance the data differences between groups. Principal Component Analysis, Pearson's correlation coefficient (PCC), multivariate analysis of variance (ANOVA), Kruskal–Wallis (KW), Recursive feature elimination (RFE), and relief feature selection were used to further screen features. The best features selected were used to build prediction models with different classifiers [including support vector machine (SVM), Auto‐Encoder (AE), Linear Discriminant Analysis (LDA), Random Forest (RF), Logistic Regression (LR), Ada‐boost (AB), Gaussian Process (GP), and Native Bayes (NB)]. The AUC was used to evaluate the performance of the model, and finally the best model was selected.

#### 2.7.2 Establishment of survival risk prediction models

The R language Survival package and Glmnet package were used for survival analysis. The Lasso_cox combination algorithm was used to screen the texture features and corresponding regression coefficients closely related to the survival time and survival state. The Rad‐score of each patient was calculated, which was a linear weight of all retained non‐zero coefficient features. The best cut‐off value of the Rad‐score was selected by X‐tile 3.6.1 (http://tissuearray.org) software, and the patients were divided into a high‐risk group and a low‐risk group according to the Rad‐score. Kaplan–Meier analysis was used to draw the survival curves of different risk groups, and *p* < 0.05 was considered statistically significant.

**FIGURE 2:**
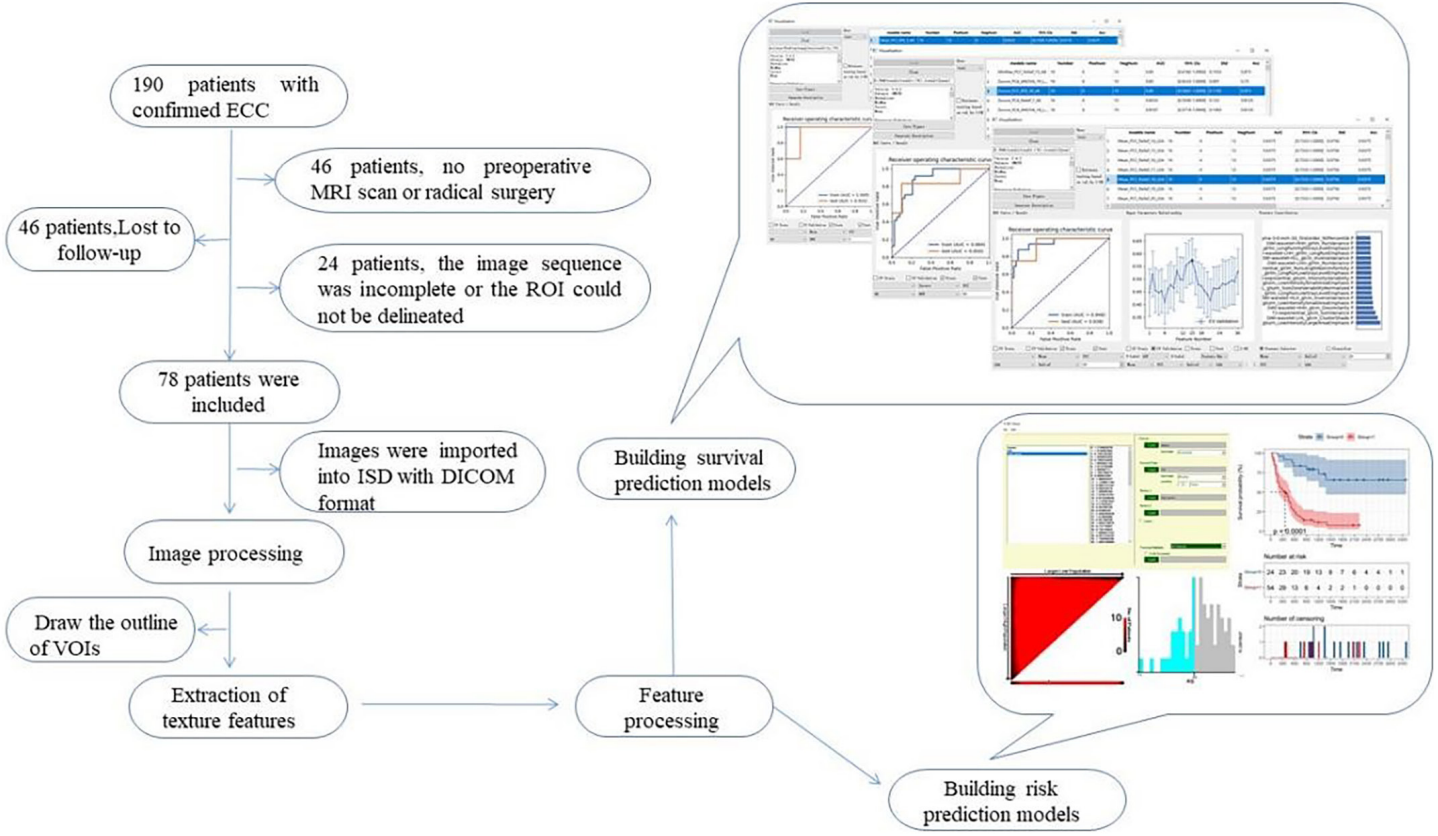
The workflow of this study.

The workflow of this study is shown in Figure 2.

## 3 RESULTS

### 3.1 Patients

**FIGURE 3:**
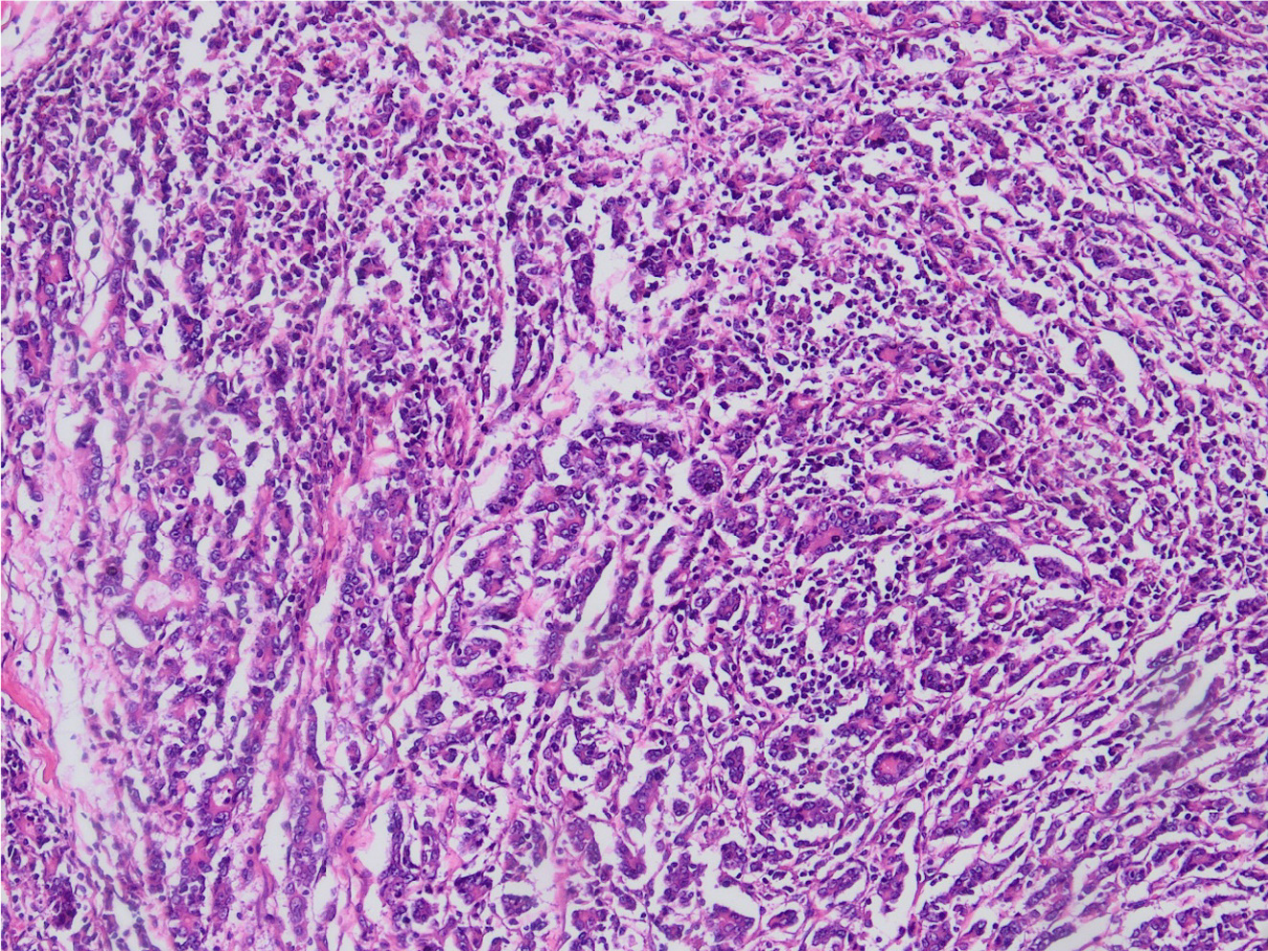
A typical pathological picture of cholangiocarcinoma.

In this study, 190 patients with ECC diagnosed by pathology in our hospital were reviewed. We excluded 46 patients because of incomplete images or no radical resection, 24 patients were excluded because the lesions were too small or showed bile duct infiltrative growth, and 42 patients were excluded because of loss to follow‐up. Finally, 78 patients were included in the study. The basic information of the patients is shown in Table 2. A typical pathological picture of cholangiocarcinoma is shown in Figure 3.

**TABLE 2:** Basic information of ECC patients.

Category	Persons/Years/Months	Ratio
Total	78	100%
Male	48	61.5%
Female	30	38.5%
Age (years)	28–79	‐
Differentiation		
High	29	37.2%
Medium	36	46.2%
Low	13	16.7%
Nerve invasion	38	48.7%
Vasculature invasion	17	21.8%
Lymph node metastasis	22	28.2%
Tumor location		
PCC	36	46.2%
DCC	42	53.8%
OS (months)	1–113	‐
Status		
Death	54	69.2%
Survival	24	30.8%
1‐year prediction model surviving	78 49	‐ 62.8%
2‐year prediction model surviving	76 30	‐ 39.5%
3‐year prediction model surviving	70 20	‐ 28.6%

*Note*: In the 1‐year prediction model, 49 patients who survived >1 year were marked as “surviving”. In the 2‐year prediction model, two ECC patients were followed up for <2 years and no death was confirmed; the sample size was thus 76 patients, of whom 30 who survived >2 years were marked as “surviving”. In the 3‐year prediction model, eight ECC patients were followed up for <3 years and no death was confirmed, resulting in a sample size of 70 patients, of whom 20 who survived >3 years were marked as “surviving”.

### 3.2 Establishment of a radiomics model based on MRI

A total of 3597 texture features were extracted from each patient. After deleting 363 features with correlation coefficients <0.75 within and between groups of ICC and nine features with null values in the feature set, 3225 texture features per patient were finally included. All characteristic values were processed by data standardization.

#### 3.2.1 Radiomics model for predicting 1‐year survival

**FIGURE 4:**
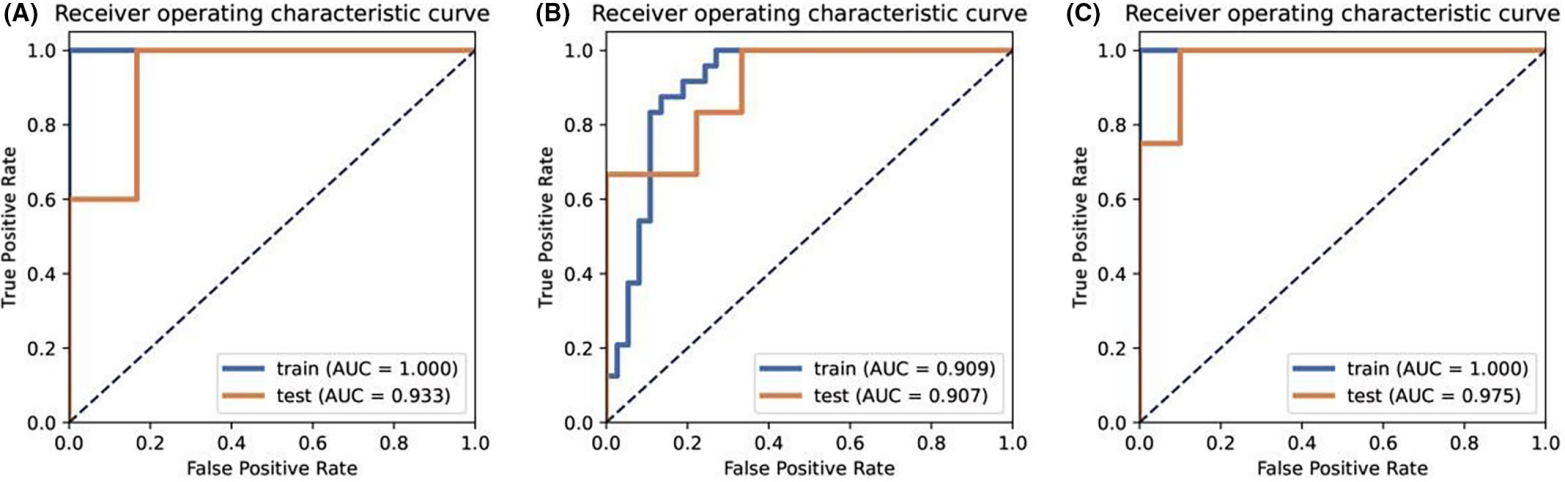
A, AUC of Mean_PCC_RFE_3_AB for predicting postoperative 1‐year survival rate. B, AUC of Mean_PCC_RFE_8_LR for predicting postoperative 2‐year survival rate. C, AUC of Z‐score_PCC_KW_25_GP for predicting postoperative 3‐year survival rate.

A total of 78 ECC patients were included. Data were balanced by SMOTE, standardized processing of mean data, reduction of redundant features by PCC, and feature selection by RFE; the prediction model was constructed using AB classifier. The three best texture features were retained to construct prediction model_a (Mean_PCC_RFE_3_AB). The AUC for predicting 1‐year survival after surgery was 1.000 in the training group and 0.933 in the test group (Figure 4A).

#### 3.2.2 Radiomics model for predicting 2‐year survival

A total of 76 ECC patients were included. Data were balanced by SMOTE, standardized processing of mean data, reduction of redundant features by PCC, and feature selection by RFE; the prediction model was constructed using LR classifier. The eight best texture features were retained to construct prediction model_b (Mean_PCC_RFE_8_LR). The AUC for predicting 2‐year survival after surgery was 0.909 in the training group and 0.907 in the test group (Figure 4B).

#### 3.2.3 Radiomics model for predicting 3‐year survival

A total of 70 ECC patients were included. Data were balanced by SMOTE, standardized processing of Z‐score, reduction of redundant features by PCC, and feature selection by KW; the prediction model was constructed using GP classifier. Finally, the 25 best texture features were retained to construct prediction model_c (Z‐score_PCC_KW_25_GP). The AUC for predicting 3‐year survival after surgery was 1.000 in the training group and 0.975 in the test group (Figure 4C).

The results of each model group are shown in Table 3.

**TABLE 3:** The best model and performance for predicting the survival rate of ECC.

	The best model	Group	AUC	Sensitivity	Specificity	PPV	NPV
Model_a	Mean_PCC_RFE_3_AB	Training test	1.000 0.933	100% 100%	100% 83.3%	100% 90.9%	100% 100%
Model_b	Mean_PCC_RFE_8_LR	Training test	0.909 0.907	87.5% 100%	86.5% 66.7%	80.8% 66.7%	71.4% 100%
Model_3	Z‐score_PCC_KW_25_GP	Training test	1.000 0.975	100% 100%	100% 90%	100% 80%	100% 100%

*Note*: Mean_PCC_RFE_3_AB was the best model for predicting the 1‐year survival rate of ECC patients, and its AUC values in the training set and test set were 1.000 and 0.933, respectively. Mean_PCC_RFE_8_LR was the best model for predicting the 2‐year survival rate of ECC patients, and its AUC values in the training set and test set were 0.909 and 0.907, respectively. Z‐score_PCC_KW_25_GP was the best model for predicting the 3‐year survival rate of ECC patients, and its AUC values in the training set and test set were 1.000 and 0.975, respectively.

#### 3.2.4 Radiomics model for predicting survival risk

**FIGURE 5:**
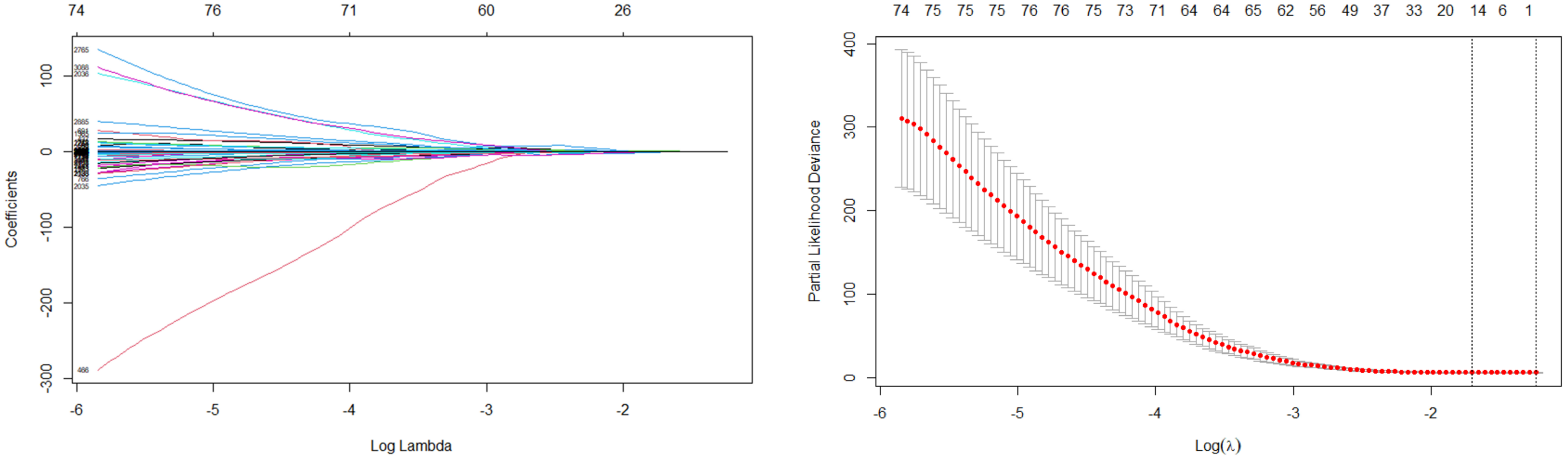
When the Lasso algorithm was executed, an increase in the λ value was correlated with a gradual decrease of the absolute value of the regression coefficient, which gradually converged to the optimal solution, and the corresponding number of selected features decreased gradually. The number of features within the range of the dotted line is the number of features that can be selected.

After generating the survival analysis dataset, the dataset was imported into the R language program workstation. The survival package and Glmnet package were used for Lasso‐cox and survival analysis processing. The Lasso‐cox algorithm selected 15 features, λ = 0.1816 (Figure 5, Table 4).

**TABLE 4:** Lasso regression screening features and corresponding regression coefficients.

Features	Regression coefficients
T1‐logarithm_glcm_Energy	−0.089
T1‐wavelet‐LHL_firstorder_Median	0.00312
T1‐wavelet‐LHH_firstorder_Skewness	0.239
T1‐wavelet‐LLH_glcm_MaximumProbability	−0.735
T1‐wavelet‐HLH_firstorder_90Percentile	0.00253
T2‐wavelet‐LHL_glcm_Imc2	−0.221
T2‐wavelet‐HLL_glcm_InverseVariance	0.611
T2‐wavelet‐LLL_glcm_ClusterShade	−0.00000123
DWI‐exponential_glszm_LowIntensityEmphasis	−0.187
DWI‐squareroot_firstorder_Skewness	0.0533
DWI‐original_glcm_InverseVariance	−0.521
DWI‐wavelet‐HLH_glcm_Energy	−0.324
DWI‐wavelet‐HLH_glrlm_LongRunEmphasis	−0.0132
DWI‐wavelet‐HLH_glrlm_LongRunLowGrayLevelEmphasis	−0.0141
DWI‐wavelet‐HHL_glszm_IntensityVariabilityNormalized	−0.708

*Note*: Lasso‐cox selected 15 texture features closely related to postoperative survival time and status of ECC patients, including five features in T1WI sequence, three features in T2WI sequence, and seven features in DWI sequence.

**FIGURE 6:**
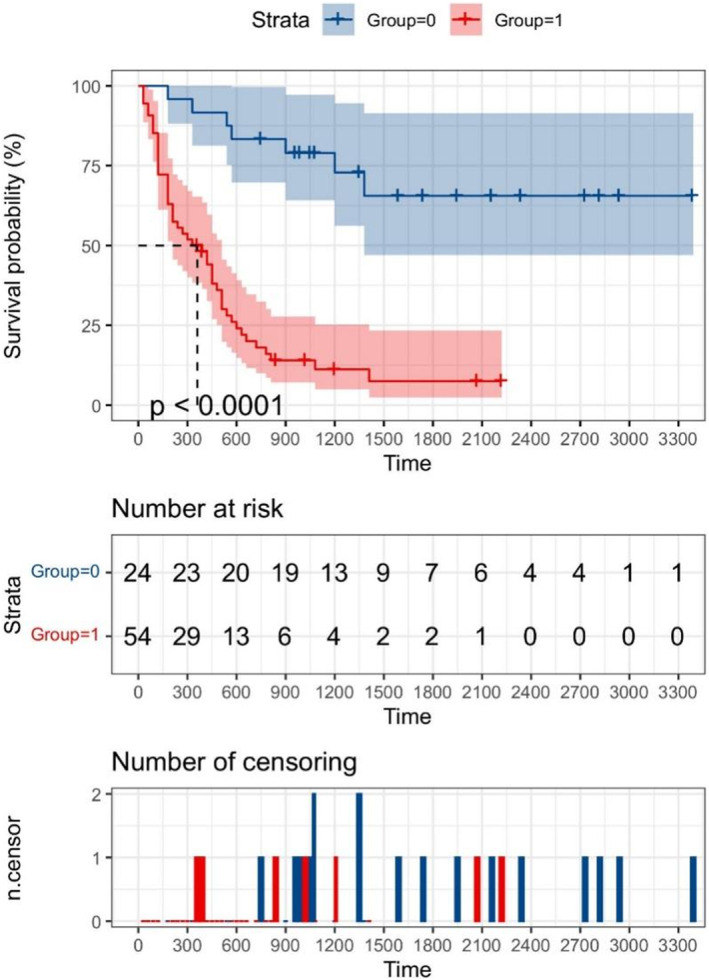
Kaplan–Meier survival analysis curve; the low risk group is designated as Group 0, and the high risk group is designated as Group 1.The unit of time is days.

X‐tile software was used to select the best cut‐off value of the Rad‐score (−0.8). According to the Rad‐score, patients with scores > − 0.8 were included in the high risk group, and those with scores <−0.8 were included in the low risk group. The details of grouping based on the Rad‐score are shown in Table 5. The Kaplan–Meier survival curve was drawn by R language survival analysis package, as shown in Figure 6. The results showed that the postoperative survival of the low‐risk group was better than that of the high‐risk group (*p* < 0.0001).

**TABLE 5:** Risk grouping based on Rad‐score.

Group	Total	Average survival time (months)	Median survival time (months)	Death	Survival
High risk	54	15.1	11.5	47(87.0%)	7(13.0%)
Low risk	24	49.1	42.5	7(29.2%)	17(70.8%)

*Note*: As of the follow‐up date, the average and median survival times of ECC patients in the high risk group after surgery were significantly lower than those of patients in the low risk group, and the number of patients who died after surgery was significantly higher than that in the low risk group. The postoperative survival was significantly lower in the high‐risk group than in the low risk group.

## 4 DISCUSSION

An increasing number of studies are focusing on assessing the survival of cancer patients. Developing methods to predict survival time before surgery is of particular importance for the design of effective treatment strategies and to improve the prognosis of patients. The purpose of this study was to explore the value of imaging in predicting the prognosis of postoperative patients with ECC, and to directly analyze and verify the correlation between imaging features and survival time and survival status. The results showed that the radiomics model could directly predict the short‐term survival rate and survival/death risk of patients with ECC.

ECC is a highly malignant primary hepatobiliary malignant tumor that affects the quality of life and survival time of patients. Without active surgical treatment, the survival time of patients is often <1 year.^13^, ^14^ Surgical resection is the first choice for the treatment of ECC, and radical resection can improve the short‐term survival rate of ECC patients.^15^ However, these studies are limited to resectable ECC patients, whereas most patients with ECC are not eligible for surgical resection at the time of disease onset. For these patients, surgical measures can accelerate the death of patients.^8^ Because the choice of treatment in different ECC patients is important to improve the prognosis, clinicians focus on the accurate assessment of patients to determine which patients can receive surgical treatment and the short‐term (1–3) survival rate after surgical treatment.

Individualized precision therapy is important for improving the prognosis of patients.^16^ The rapid development of traditional imaging methods such as CT, PET/CT, and MRI among others has markedly improved the diagnosis of ECC, and the evaluation of preoperative resectability of ECC has also reached a more accurate level.^17^, ^18^, ^19^ However, the traditional imaging examination is subjective and affected by many factors. In some patients with ECC who are considered resectable before undergoing surgery, lymph node metastasis or peritoneal invasion are found during the operation, which leads to an ineffective operation^20^ that may worsen the prognosis of the patient. Advances in imaging science have improved the design of individualized and accurate therapies. The basic theory of imaging science is to extract features that cannot be recognized by the naked eye using high‐throughput extraction, and to find the correlation between different features and cellular and molecular pathological changes using statistical methods. The purpose is to obtain information on tumor pathology and prognosis from the image itself.

The most commonly used survival analysis method is the Kaplan–Meier method for drawing survival curves. This method is combined with variables, survival time, and survival status for analysis, and the results of the survival curve evaluation determine the statistically significant differences between groups. The radiomics score is based on the high‐throughput texture feature parameters extracted by image group. In the Lasso regression algorithm, a feature screening method, some of the most representative texture features are retained, and their regression coefficients are calculated. Finally, through the use of specific formulas, the Rad‐score of each patient can be calculated, and the image information is transformed into specific numerical information. The application of the Rad‐score has achieved good results in recent years. For example, Jiang et al.^21^ divided patients with gastric cancer into high and low risk groups according to the 5‐year survival and disease‐free survival using 18F‐PET/CT‐based image features to calculate imaging tags. The results showed that patients with high Rad‐scores were more likely to benefit from chemotherapy. Meng et al.^22^ applied imaging labels to divide advanced rectal cancer patients into high and low risk groups based on disease‐free survival. The results showed that there were significant differences between groups. Multivariate regression analysis showed that the Rad‐score was an independent risk factor for predicting disease‐free survival in patients with advanced rectal cancer, and combined with clinical models, it could significantly improve the ability to predict disease‐free survival.

In this study, the machine learning imaging method was used to construct an imaging model based on the postoperative survival time of patients with ECC. The results showed that the imaging model has a good ability to predict the survival time of ECC patients. The model predicted the 1‐year survival rate after ECC diagnosis, with AUC values in the training group and the test group of 1.000 and 0.933, respectively. The AUC values of the model in the training group and the test group were 0.909 and 0.907, respectively, for predicting 2‐year survival, and 1.000 and 0.975, respectively, for predicting 3‐year survival. In addition, the risk grouping model of ECC patients was constructed by imaging, and the results of Kaplan–Meier survival analysis showed that there was a significant difference in the risk of survival/death among different risk groups (*p* < 0.0001). Machine learning analysis of preoperative images of ECC patients can directly predict the postoperative survival of ECC patients, which is important for the choice of clinical treatment. For example, for ECC patients whose predicted postoperative survival time is <1 year or the risk score places them in the high risk group, palliative support treatment instead of traumatic surgical treatment will improve the prognosis and reduce the financial burden of patients to some extent.

This study had several limitations. First, the included cases were single‐center samples, and multicenter comparative studies were not performed. Second, the differences in cholangiocarcinoma lesions (except subtypes of mass type) made it difficult to accurately identify the lesions and outline the ROI, and the exclusion of some cases may lead to selection bias. Finally, the number of patients lost to follow‐up decreased the sample size in this study.

## 5 CONCLUSION

The imaging model based on MRI could predict the survival and prognosis of patients with ECC and guide the clinical choice of appropriate treatments.

## AUTHOR CONTRIBUTIONS

**Limin Wang:** Conceptualization (lead); investigation (equal); methodology (equal); resources (lead); software (lead); writing – original draft (lead). **Jiong Liu:** Data curation (supporting); investigation (equal); methodology (equal); resources (supporting); software (supporting); visualization (supporting). **Yanyan Zeng:** Data curation (supporting); investigation (equal); methodology (equal); resources (supporting); supervision (equal). **Jian Shu:** Conceptualization (equal); funding acquisition (lead); methodology (equal); project administration (lead); resources (supporting); supervision (lead); writing – review and editing (equal).

## FUNDING INFORMATION

This study has received funding by Key projects of Science and Technology Department of Sichuan Province (No. 2022YFS0070).

## ETHICS STATEMENT

Our hospital ethics committee (Clinical Trial Ethics Committee of the Affiliated Hospital of Southwest Medical University) approved this retrospective study and waived patient informed consent(KY2023041).

## Data Availability

The data that support the findings of this study are available from the corresponding author upon reasonable request.
